# The Effectiveness of Mindfulness-Based Relapse Prevention on Chinese Methamphetamine Dependent Patients: A Pilot Study

**DOI:** 10.3389/fpsyt.2022.819075

**Published:** 2022-02-28

**Authors:** Jing Zhai, Yan Long, Jingqing Shi, Daqing Shi, Qihuan Ren, Min Zhao, Jiang Du

**Affiliations:** ^1^Shanghai Mental Health Center, Shanghai Jiao Tong University School of Medicine, Shanghai, China; ^2^Shanghai Key Laboratory of Psychotic Disorders, Shanghai Jiao Tong University School of Medicine, Shanghai, China; ^3^Chinese Academy of Sciences (CAS) Center for Excellence in Brain Science and Intelligence Technology (CEBSIT), Chinese Academy of Sciences, Shanghai, China

**Keywords:** mindfulness, intervention, China, methamphetamine, dependence

## Abstract

Methamphetamine use is a serious problem in China. Compulsory isolation detoxification is the main treatment measure for drug dependents, whereas psychological interventions in compulsory isolation detoxification centers are extremely inadequate. The current study aimed to examine the effects of mindfulness-based relapse prevention (MBRP) on methamphetamine dependence patients in Chinese compulsory isolation detoxification treatment institutions. Forty-one methamphetamine dependent patients received 16-sessions of MBRP in 8 weeks and assessments were conducted at the baseline, 4-, 8-week (after the whole intervention). Results of repeated measured ANOVAs showed there was no significant effect on emotions and cravings. Findings indicated that the effects of MBRP are still difficult to make firm conclusions due to the insignificant results. Future studies should modify the MBRP and ensure that it is suitable for compulsory isolation detoxification treatment institutions in China.

## Introduction

Substance use is a serious public health problem and social problem. According to a report by the Chinese government, the number of drug users in China has reached 1.801 million in 2020, 57.2% of them abuse synthetic drugs such as methamphetamine and ketamine, and methamphetamine abuse remains a serious problem (1). Compared to traditional drugs, methamphetamine showed higher addictive and neurotoxicity which would cause brain dysfunction, psychotic symptoms, cognitive impairment, and high risks of relapse (2). Methamphetamine dependent patients generally showed higher craving, impulsive, and negative emotions, and worse cognitive functions (3–5).

In China, compulsory isolation detoxification is the main treatment measure for drug dependents. Compared with the previous treatment system, compulsory isolation detoxification addresses the importance of psychological intervention among the drug dependents since according to the “Drug Control Law of the People's Republic of China,” drug dependents have been defined as both offenders and patients, and psychological interventions should be delivered to help them to keep abstinence. To meet the requirement of “Drug Control Law,” we try to find new psychological intervention strategies which are eligible for compulsory isolation treatment sites.

Mindfulness-based relapse prevention therapy (MBRP) developed by Witkiewitz et al. (6) is one of the psychotherapies targeted at substance use disorders (SUDs). MBRP integrated mindfulness meditation and traditional relapse prevention and aimed to help participants develop a sustainable lifestyle and utilize mindfulness skills as effective coping strategies in the face of high-risk situations to prevent relapse (6).

The effect of MBRP on SUDs has received considerable empirical support (7). Compared to the control group, MBRP could reduce patients' craving for drugs and improve their ability to control their negative emotions which is the high-risk factor for relapse. But whether this intervention is appropriate for Chinese compulsory treatment institutions has not been verified.

The pilot study aimed to examine the effectiveness of MBRP on methamphetamine dependent patients in Chinese compulsory isolation detoxification treatment institutes.

## Methods

### Participants

Forty-one participants were recruited from the compulsory isolation detoxification treatment center in < city>Shanghai < /city>, China from April to May in 2019. The inclusion criteria were: (1) met the diagnosis of amphetamine-type substance use disorder in the Diagnostic and Statistical Manual of Mental Disorders 5 (DSM-5); (2) completed physiological detoxification; (3) capable of independent communication; (4) aged 18–60. All participants signed informed consent before entering the study.

### Procedure

**Figure 1:**
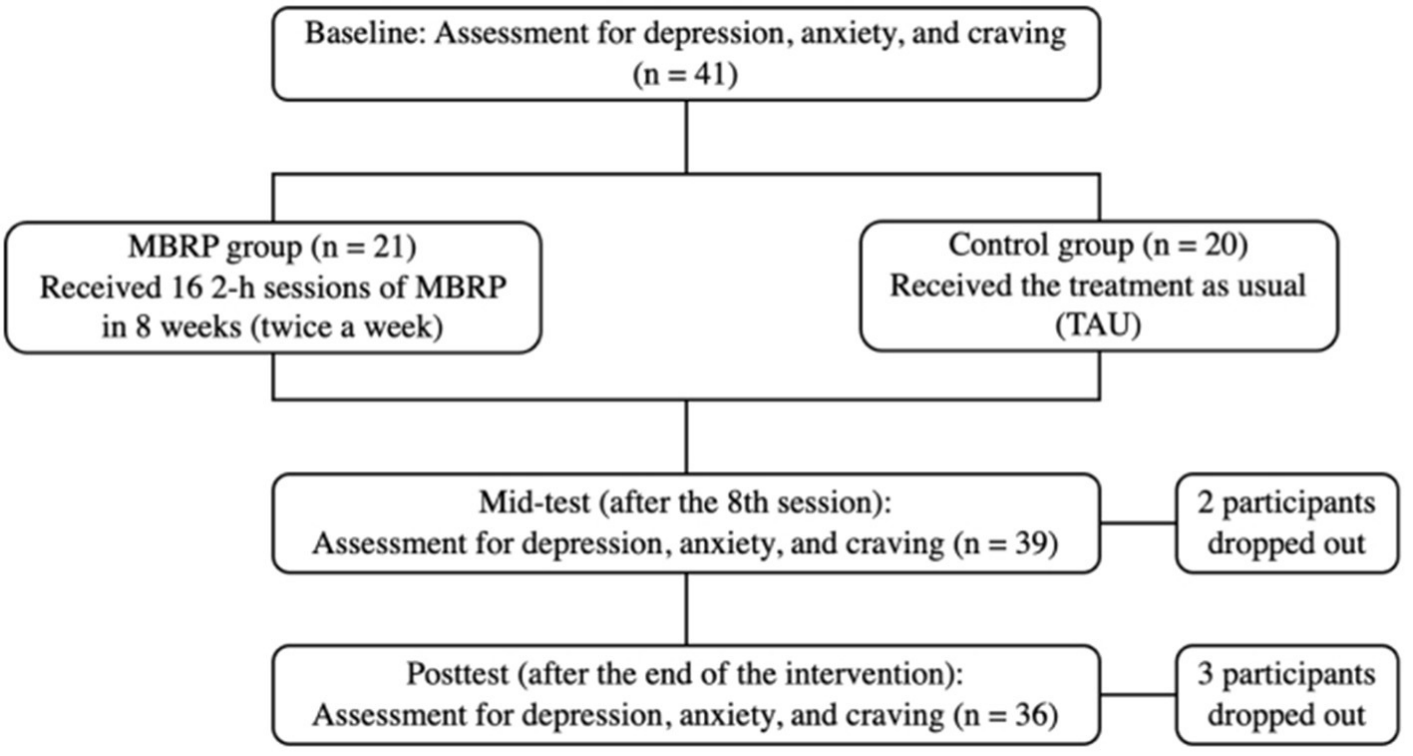
The flow chart of the current study.

**Figure 2:**
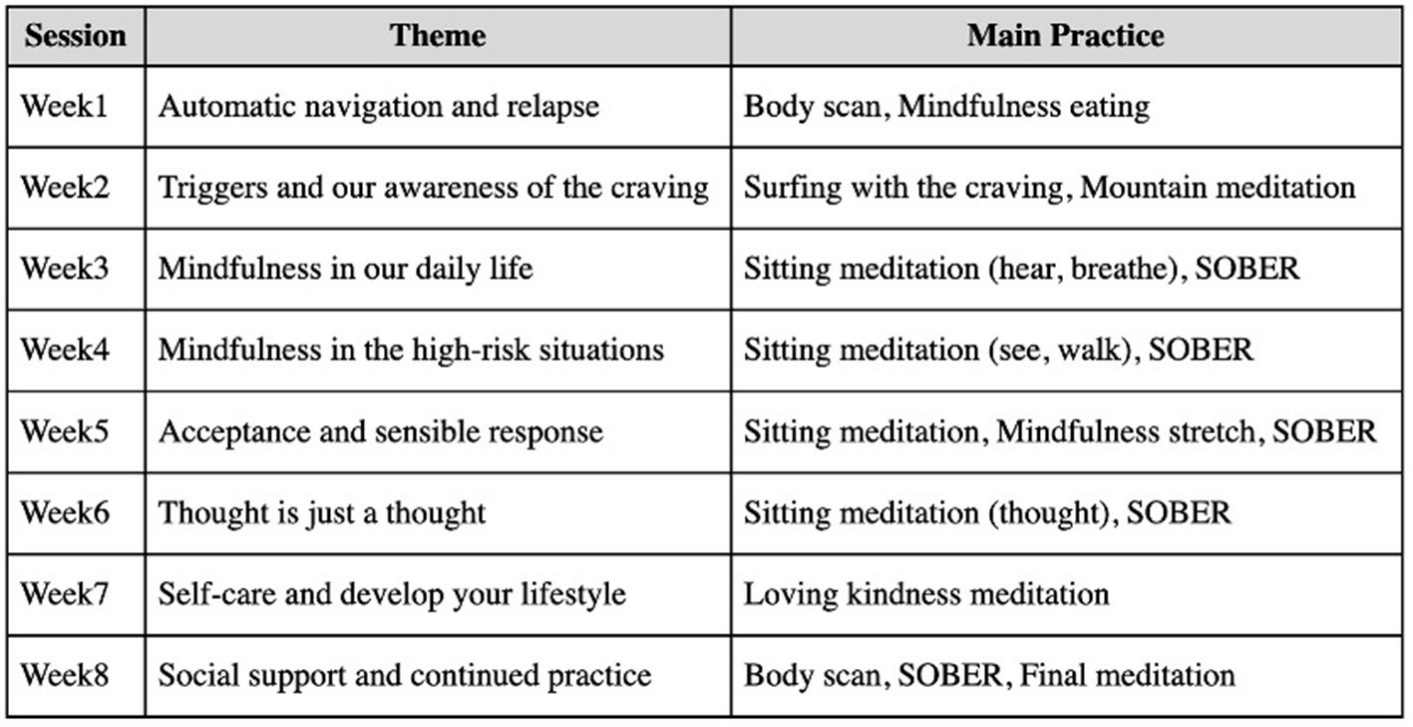
The main content of each intervention sessions.

This study was designed as a randomized controlled clinical trial and has been registered at ClinicalTrials.gov (ID: NCT03748875). Forty-one participants were randomly divided into the MBRP group (*n* = 21) and the control group (*n* = 20). The MBRP group received 16 sessions of 2-h MBRP interventions (8 weeks, twice per week), which was developed based on the *Mindfulness-based relapse prevention for addictive behaviors: a clinician guide* written by Bowen et al. The intervention was led by a professional mindfulness trainer. The control group received the treatment as usual (TAU). Assessments were conducted at the baseline, after the 8th session (mid-test), and the end of the intervention (post-test). See Figure 1 for the flow chart of the current study and Figure 2 for the detailed content of the intervention.

### Assessment

#### Depression

The Chinese version of the Beck Depression Inventory (BDI) was used to measure depression (8). This scale consists of 21 items scored on a 4-point Likert scale, ranging from 0 to 3. A higher score represents a higher level of depression. The psychometric properties of BDI were acceptable in Chinese samples (8).

#### Anxiety

Participants' anxiety was measured using the Chinese version of the State-Trait Anxiety Inventory (STAI) (9). This scale consists of 40 items scored from 1 to 4, contains two sub-scales: state anxiety and trait anxiety. A higher score indicates a higher level of anxiety. The Chinese version of STAI has satisfying psychometric properties (9).

#### Craving

Craving was measured using the Visual Analog Scale (VAS). The 100 mm line was used to represent the craving degree from 0 (no craving) to 100 (very strong craving). A higher score indicates a higher level of craving.

### Data Analysis

Data were processed with SPSS 21.0. χ^2^ test and independent *t*-test were used to examine group differences at the baseline. Emotions and craving measured at the baseline, mid-test, and post-test were submitted to 3 (Time) × 2 (Group) repeated measures ANOVAs to examine the effects of the intervention. The significant level was set at 0.05.

## Results

### Baseline Characteristics

The results of the χ^2^-test and independent *t*-tests indicated no significant difference between the two groups at the baseline except depression (*p*s > 0.05). The BDI scores in the MBRP group were significantly higher than that in the control group at the baseline [*t*_(39)_ = 2.88, *p* = 0.006]. Thus, depression was regarded as a covariate in the following analyses. See Table 1 for more details.

**Table 1:** Baseline comparison of demographic variables, substance use situations, and all outcome variables between the MBRP group and the control group before the intervention [*M* ± *SD/n(%)*] (*N* = 41).

	**MBRP group** **(*n* = 21)**	**Control group** **(*n* = 20)**	**χ** ^**2**^ **/t**	***p***
Age	35.38 ± 6.045	38.80 ± 9.395	−1.393	0.172
**Marriage**	0.299	0.585		
Married	8 (38.1%)	6 (30.0%)		
Single or divorced	13 (61.9%)	14 (70.0%)		
Years of education	11.05 ± 3.43	12.40 ± 2.96	−1.349	0.185
**Occupation**	0.811	0.368		
Employed	13 (61.9%)	15 (75.0%)		
Unemployed	8 (38.1%)	5 (25.0%)		
Months used accumulatively	29.88 ± 49.67	37.21 ± 54.94	−0.443	0.660
**Compulsory isolation times**	0.786	0.675		
Once	16 (76.2%)	13 (65%)		
Twice or three times	3 (14.3%)	5 (25%)		
More than three times	2 (9.5%)	2 (10%)		
BDI	19.29 ± 7.23	13.10 ± 6.49	2.878^*^	0.006
**STAI**				
State anxiety	40.62 ± 9.368	42.15 ± 8.63	−0.543	0.590
Trait anxiety	44.24 ± 12.03	40.75 ± 8.78	1.056	0.297
VAS craving	28.67 ± 27.20	20.3 ± 26.26	1.001	0.323

* *p < 0.05*.

### Intervention Effects

Five participants dropped out because of private reasons. The remaining data from 36 participants were included in further analyses. The results of repeated measured ANOVAs showed that the main effects of group on BDI and state anxiety were significant [*F*_(1, 34)_ = 4.890, *p* = 0.034, $\eta_{\text{p}}^{2}$ = 0.126, *power* = 0.575; *F*_(1, 34)_ = 4.445, *p* =.043, $\eta_{\text{p}}^{2}$ = 0.119, *power* = 0.535]. The further pairwise comparison showed that the BDI scores in the MBRP group were significantly higher than that in the control group (*p* = 0.034), the score of state anxiety in the MBRP group was significantly lower than that in the control group (*p* = 0.043). Moreover, time showed a significant main effect on BDI [*F*_(2, 34)_ = 5.271, *p* = 0.007, $\eta_{\text{p}}^{2}$ = 0.134, *power* = 0.819]. The BDI scores at the post-test were significantly lower than that at the baseline (*p* = 0.009) and the mid-test (*p* = 0.020). However, no significant interaction effects of time and groups were found (*p*s > 0.05). See more details in Table 2.

**Table 2:** The results of the repeated measured ANOVAs in all outcome variables (*N* = 36).

		**MBRP group** **(*n* = 20)**	**Control group** **(*n* = 16)**	**Repeated-Measured ANOVAs**				
					***F***	***p***	***η** ^**2**^*	***Power***
BDI	Baseline	19.30 ± 7.42	12.94 ± 6.75	G	4.89^*^	0.034	0.126	0.575
	Mid-Test	16.60 ± 7.82	13.19 ± 6.86	T	5.271^*^	0.007	0.134	0.819
	Post-test	14.90 ± 9.09	10.12 ± 7.00	G × T	0.854	0.43	0.025	0.191
**STAI**								
State anxiety	Baseline	40.85 ± 9.55	43.125 ± 9.14	G	4.445^*^	0.043	0.119	0.535
	Mid-Test	41.10 ± 9.23	43.875 ± 12.17	T	1.592	0.211	0.046	0.326
	Post-test	40.30 ± 10.62	43.75 ± 12.87	G × T	0.710	0.495	0.021	0.165
Trait anxiety	Baseline	44.40 ± 12.31	41.4375 ± 9.37	G	2.365	0.134	0.067	0.321
	Mid-Test	42.15 ± 9.89	42.44 ± 10.70	T	1.050	0.356	0.031	0.226
	Post-test	41.8 ± 10.78	42.19 ± 9.11	G × T	1.076	0.347	0.032	0.231
VAS	Baseline	29.85 ± 27.34	19.063 ± 25.91	G	0.358	0.554	0.011	0.089
	Mid-Test	30.05 ± 30.72	20.19 ± 30.549	T	1.320	0.274	0.038	0.276
	Post-test	21.65 ± 25.29	16.56 ± 23.301	G × T	0.714	0.494	0.021	0.166

* *G, group; T, time; p < 0.05*.

*The reviewer CZ declared a shared affiliation, with no collaboration, with the authors JZ, YL, JS, DS, QR, MZ, and JD at the time of the review*.

## Discussion

This pilot study initially examined the effectiveness of MBRP on methamphetamine dependent patients in Chinese compulsory isolation detoxification treatment institutes. We focused on the effects of MBRP on patients' emotions and cravings in the present study.

The main effect of time on BDI is significant, which indicated that the depression level of methamphetamine dependent patients declined over time. Previous studies found that in the rehabilitation period, the depression level of methamphetamine dependent patients improved during the time course, which was consistent with the current findings (10). Most notably, the interactions between time and group were not significant on emotions and craving. These results indicated that MBRP showed no significant improved effects on patients' depression, anxiety, and craving. Previous studies generally suggested that MBRP is effective for SUDs to improve emotions and reduce cravings (11). Glasner et al. (12) proved that MBRP could significantly reduce stimulant abuse patients' depression. Similarly, Zullig et al. (13) found that MBRP showed considerable effects on improving opioid use patients' anxiety.

However, numerous studies have suggested that the MBRP intervention has no significant effects on the improvement of emotions and cravings among SUDs. Previous research observed no significant effects of MBRP on anxiety and craving (13). According to Zullig et al. (13), current results may be at least partially explained by the goals of MBRP are to increase the awareness of cravings and reduce behavioral and emotional reactivity to cravings, but not to eliminate the cravings themselves.

Regarding the current study, it was conducted in the compulsory isolation detoxification treatment institutes, in which patients' daily schedules were strictly arranged. Thus, the lacking meditation practice might reduce the intervention effect. Another impacting factor is the changing motivation. The participants in the compulsory isolation detoxification treatment institutes are relatively lacking in changing motivation, which might be an important reason for the insignificant effect of MBRP intervention in the current study (14). Other factors include small sample size, gender bias (all males), which will affect the research findings to a certain extend. More importantly, previous studies found that methamphetamine showed higher neurotoxicity and addiction (2), which might also affect the effectiveness of the MBRP intervention. Further studies could conduct in a larger sample size and combine other techniques (such as virtual realit or motivation intervention) with MBRP to improve the effectiveness of MBRP in methamphetamine dependent patients.

Even the results were negative, the present study still provides preliminary evidence for the implementation of MBRP on methamphetamine patients. Given the specific settings for compulsory rehabilitation treatment in China, it might be necessary to modify MBRP and ensure it is suitable for the current situation.

Limitations to this study include assessing all data *via* self-report scales which might be impacted by social desirability bias, all the included methamphetamine dependent patients are male while gender might influence the vulnerability to methamphetamine toxicity, and lack of biological indicators as the biological improvements might not be reflected in behaviors (15, 16). It would be worthwhile to conduct further study in a larger sample using comprehensive measurements, such as the behavioral tasks to expand the result.

## Data Availability Statement

The raw data supporting the conclusions of this article will be made available by the authors, without undue reservation.

## Ethics Statement

The studies involving human participants were reviewed and approved by the Institutional Review Boards of the Shanghai Mental Health Center. The patients/participants provided their written informed consent to participate in this study.

## Author Contributions

JD and MZ contributed to the conceptualization and design of the study. JS, DS, and QR collected the data. YL performed the statistical analysis and the interpretation of data. JZ and YL drafted the manuscript. JD provided scientific revisions to the manuscript. All authors contributed to the article and approved the submitted version.

## Funding

This work was supported by National Nature Science Foundation of China (81871045 and 8217050343), Science and Technology Commission of Shanghai Municipal (19411969200 and 21DZ2201000), Shanghai Municipal Science and Technology Major Project (2018SHZDZX05), Shanghai Clinical Research Center for Mental Health (19MC1911100), Shanghai Engineering Research Center of Intelligent Addiction Treatment and Rehabilitation (19DZ2255200), and Shanghai Key Laboratory of Psychotic Disorders (13DZ2260500).

## Conflict of Interest

The authors declare that the research was conducted in the absence of any commercial or financial relationships that could be construed as a potential conflict of interest. The reviewer CZ declared a shared affiliation, with no collaboration, with the authors JZ, YL, JS, DS, QR, MZ, and JD at the time of the review.

## Publisher's Note

All claims expressed in this article are solely those of the authors and do not necessarily represent those of their affiliated organizations, or those of the publisher, the editors and the reviewers. Any product that may be evaluated in this article, or claim that may be made by its manufacturer, is not guaranteed or endorsed by the publisher.
